# Genetic Testing in Hereditary Breast and Ovarian Cancer Using Massive Parallel Sequencing

**DOI:** 10.1155/2014/542541

**Published:** 2014-06-26

**Authors:** Anna Ruiz, Gemma Llort, Carmen Yagüe, Neus Baena, Marina Viñas, Montse Torra, Anna Brunet, Miquel A. Seguí, Eugeni Saigí, Miriam Guitart

**Affiliations:** ^1^Laboratorio de Genética, UDIAT-Centre Diagnòstic, Corporació Sanitària Parc Taulí Institut Universitari (UAB), Parc Taulí s/n, Sabadell, 08208 Barcelona, Spain; ^2^Unidad de Consejo Genético, Institut Oncològic del Vallès, Corporació Sanitària Parc Taulí, Parc Taulí s/n, Sabadell, 08208 Barcelona, Spain; ^3^Departamento de Investigación Oncológica, Corporació Sanitària Parc Taulí Institut Universitari (UAB), Parc Taulí s/n, Sabadell, 08208 Barcelona, Spain; ^4^Departamento de Oncología Médica, Corporació Sanitària Parc Taulí Institut Universitari (UAB), Parc Taulí s/n, Sabadell, 08208 Barcelona, Spain

## Abstract

High throughput methods such as next generation sequencing are increasingly used in molecular diagnosis. The aim of this study was to develop a workflow for the detection of *BRCA1* and *BRCA2* mutations using massive parallel sequencing in a 454 GS Junior bench top sequencer. Our approach was first validated in a panel of 23 patients containing 62 unique variants that had been previously Sanger sequenced. Subsequently, 101 patients with familial breast and ovarian cancer were studied. *BRCA1* and *BRCA2* exon enrichment has been performed by PCR amplification using the BRCA MASTR kit (Multiplicom). Bioinformatic analysis of reads is performed with the AVA software v2.7 (Roche). In total, all 62 variants were detected resulting in a sensitivity of 100%. 71 false positives were called resulting in a specificity of 97.35%. All of them correspond to deletions located in homopolymeric stretches. The analysis of the homopolymers stretches of 6 bp or longer using the BRCA HP kit (Multiplicom) increased the specificity of the detection of *BRCA1* and *BRCA2* mutations to 99.99%. We show here that massive parallel pyrosequencing can be used as a diagnostic strategy to test for *BRCA1* and *BRCA2* mutations meeting very stringent sensitivity and specificity parameters replacing traditional Sanger sequencing with a lower cost.

## 1. Introduction

Germline mutations that inactivate* BRCA1* and* BRCA2* are responsible for breast and ovarian cancer susceptibility [1, 2]. The prevalence of* BRCA1* and* BRCA2* mutations where family history shows more than one occurrence of breast cancer under the age of 50 ranges from 8 to 21.2%. Mutation carriers are at an increased cumulative risk to the age of 70 of 36–70% and 10–65% for breast cancer and ovarian cancer, respectively [3, 4]. Moreover,* BRCA1* and* BRCA2* mutation carriers are also at increased risk of pancreatic, prostate, and endometrial cancer. Molecular diagnosis is an important factor in clinical decisions that include increased surveillance, chemoprevention, or prophylactic surgery [5, 6]. Predictive testing in family members allows the identification of other individuals at risk.

*BRCA1* and* BRCA2* mutation screening is offered to patients from high risk families. Direct Sanger sequencing allows the identification of the sequence alteration and is considered the gold standard. Sequencing of* BRCA1* and* BRCA2* genes is time consuming and costly due to the large size of the genes and the equal distribution of mutations along the whole* BRCA1* and* BRCA2* sequence (5589 and 10254 nucleotides, resp.). A high level of allelic heterogeneity has been described including single nucleotide variants (SNVs), short insertions and deletions (InDels), and large structural variants (see Breast Cancer Information Core database: http://www.research.nhgri.nih.gov/bic/). Currently, many laboratories include a scanning method that allows the detection of all different types of mutations with a sensitivity and specificity of 100% [7].

High throughput methods such as next generation sequencing are increasingly used in molecular diagnosis [8]. Massive parallel sequencing allows the generation of millions of DNA sequences in a single run with low cost per base [9]. The development of technologies to capture and enrich specific regions of the genome improves performance and reduces the cost allowing joint sample analysis of numerous individuals [10]. Several studies have demonstrated the potential of massive sequencing both in the field of research and in genetic diagnosis [11, 12]. Recently, next generation sequencing methods for the mutation analysis of the* BRCA1* and* BRCA2* genes in patients with breast and ovarian cancer have been described using both high capacity and bench top platforms [13–18]. Bench top sequencers are addressed to individual labs to suit the demand of midsize diagnostic laboratories.

Here, we developed a workflow using massive parallel pyrosequencing in a bench top 454 GS Junior sequencer together with homopolymer scanning to screen for mutations in the* BRCA1* and* BRCA2* genes. Our workflow was first validated in a panel of 23 patients previously Sanger sequenced. Subsequently, 101 patients with familial breast and ovarian cancer were studied. We found 18 pathogenic mutations and 10 variants with unknown clinical significant effect (VUS). We show here that our workflow performs as Sanger sequencing in terms of sensitivity and specificity with the advantage of taking less time and cost consuming being suitable for genetic diagnosis.

## 2. Methodology

### 2.1. Patients

A total of 23 samples containing 62 unique variants were used to evaluate the methodology. 49 variants corresponded to single nucleotide variants (SNV) while 13 corresponded to deletions (8), insertions (3), and combined insertions and deletions (2). Among the 62 variants tested 14 were pathogenic mutations (11 insertions/deletions, 1 missense mutation, 1 nonsense mutation, and 1 splice site mutation). DNA samples were obtained from the Hereditary Cancer Program at the Catalan Institute of Oncology (ICO-IDIBELL) and the Genetic Counselling Unit at the Hospital of Sabadell (Barcelona, Spain).

Then, 101 patients with breast and ovarian cancer were screened for mutations using our validated workflow. DNA samples were collected from patients referred to the Genetic Counselling Unit at the Hospital of Sabadell (Barcelona, Spain). Informed consent was obtained from all the patients included in our study. Genomic DNA was extracted from peripheral blood following standard procedures and using Gentra Puregene DNA reagents (Qiagen, Valencia, CA, USA).

### 2.2. Multiplex PCR Target Amplification, NGS Library Preparation, and Sequencing

*BRCA1* and* BRCA2* coding regions and exon intron boundaries were amplified using the BRCA MASTR kit (Multiplicom, Niel, Belgium). Samples used to evaluate the methodology performance were amplified using the BRCA MASTR kit v1.2 (7 samples) and v2.0 (16 samples) following manufacturer instructions. The samples screened for* BRCA1* and* BRCA2* mutations were amplified using the BRCA MASTR kit v2.1. The BRCA MASTR kit v1.2 amplifies* BRCA1* and* BRCA2* coding regions and exon intron boundaries in 169 amplicons while versions 2.0 and 2.1 amplify both genes in 94 and 93 amplicons, respectively. Briefly, 50 ng of genomic DNA was used in a two-step multiplex reaction to firstly amplify* BRCA1* and* BRCA2* coding regions followed by the incorporation of molecular barcodes (multiple identifiers, MIDs) and 454 adapters to each amplicon. A* BRCA1* and* BRCA2* amplicon library of each patient was generated and quantified using Quant-iT PicoGreen (Invitrogen, Life Technologies, San Diego, CA, USA). Equivalent amounts of the patient libraries were pooled to generate a unique sequencing library that is twice purified using Agencourt AMPure XP (Beckman Coulter, Beverly, MA, USA) and PicoGreen quantified. Emulsion PCR was performed using the GS Junior Titanium emPCR kit (Lib-A) and pyrosequenced in the sense and antisense strands with the GS Junior following manufacturer's instructions (Roche Applied Science, Mannheim, Germany).

### 2.3. Bioinformatic Analysis

Data analysis was performed using the GS Amplicon Variant Analyzer version 2.7 (AVAv2.7) software (Roche). After sequence quality filtering, specific primers, MIDs, and adapter sequences are trimmed. Reads are then mapped to* BRCA1* and* BRCA2* genomic reference sequences NG_005905 and NG_012772, respectively. Coverage was obtained for all amplicons and analysed to detect low coverage amplicons. Variants are filtered using the AVAv2.7 software according to two parameters, the presence of the variant in both strands and the percentage of reads with the variant. Finally, variants are annotated according to the Human Genome Variation Society guidelines (http://www.hgvs.org/). Functional significance of variants is assigned by the authors following established criteria [19].

### 2.4. Homopolymer Analysis

*BRCA1* and* BRCA2* coding homopolymers of 6 bp or longer were analyzed using the BRCA HP v.2.0 (Multiplicom). Briefly, 50 ng of genomic DNA is amplified in two multiplex reactions resulting in 39 fragments that comprise all coding homopolymers. Fragment length is analysed on the ABI 3130 sequencer using the Gene Mapper software (Applied Biosystems, Foster City, CA, USA).

### 2.5. Multiple Amplicon Quantification (MAQ) Analysis

*BRCA1* and* BRCA2* large rearrangements were analysed using the BRCA MAQ kit (Multiplicom). It consists in the simultaneous amplification of several fluorescently labelled target amplicons (*BRCA1* and* BRCA2* exons) and reference sequences. Fragments are then size separated on an ABI 3130 sequencer (Applied Biosystems). Comparison of the relative intensities of the target amplicons in the test individual and a control individual results in a dosage quotient, indicating the copy number of the CNV in the test sample.

## 3. Results

### 3.1. Validation of Next Generation Sequencing Performance for* BRCA1* and* BRCA2* Mutation Screening

In order to evaluate our massive parallel sequencing approach 23 patients previously Sanger sequenced were pyrosequenced in a 454 GS Junior platform.

In total three runs were performed. In the first run 7 samples were sequenced using the BRCA MASTR kit v1.2 while in the last two runs 8 samples were simultaneously sequenced using the BRCA MASTR kit v2.0. The number of reads was variable between the runs. The average coverage per amplicon was higher in the third run. The use of the BRCA MASTR kit v2.0 that amplifies the two BRCA genes in 94 amplicons instead of 169 increases the average number of reads per amplicon as well as decreasing the number of amplicons with less than 38 reads even though in the first run we sequenced seven samples instead of eight (Table 1).

Bioinformatic analysis of reads is performed with the AVA software v2.7 (Roche). First, adapter and MIDs are trimmed from the obtained reads. Then, reads are mapped to the references sequences and variants are called and reported. We considered true variants those found in both strands and present in at least 25% of reads. The list of variants reported by the AVA software was further filtered excluding those variants present in amplicons with less than 38x coverage. It has been described that a minimum coverage of 38x is required to obtain a Phred score of 30 (or *P* = 99.9%) when using a variant detection filter of 25% [20]. The number of amplicons with less than 38x coverage ranged from 13 to 32 which represent less than 5% of the total number of amplicons sequenced. All 49 distinct substitutions were detected both in heterozygosity and homozygosity. Heterozygous substitutions were detected between 25% and 76.47% of the reads while homozygous substitutions were detected between 97.44% and 100% of the reads (Supplementary Table 1 availabe online at http://dx.doi.org/10.1155/2014/542541). As expected the variant detection is closer to 50% with high coverage. All the deletions and insertions, except from c.548-58delTT located in a homopolymeric stretch of 7 T in intron 7 of the* BRCA1* gene, were detected in both the forward and the reverse strands and between 26% and 82.14% of the reads. We detected the variant c.548-58delTT in all samples at high frequency even though it was not present in all samples resulting in a false positive. Deletion of c.6841+79delTTAA in intron 11 of the* BRCA2* gene was detected both in heterozygosity and homozygosity. The pathogenic variants were all detected in heterozygosity (Table 2) except for the c.8946delA in the* BRCA2* gene which was detected in homozygosity in the forward reads. This is due to the location of the c.8946delA in a homopolymer stretch. In total, all 62 variants were detected resulting in a sensitivity of 100%.

We detected 37 different false positives with the AVA software 2.7 (Supplementary Table 2). All of them correspond to deletions located in homopolymeric stretches and are generated as a result of the use of the pyrosequencing technology as has been described previously [21]. 35 out from 37 correspond to deletions in homopolymeric stretches of 6 bp or longer. The remaining 2 false positives correspond to two deletions at homopolymers of 4 nucleotides. In the total of three runs 71 false positives were called resulting in a specificity of 97.35%. The analysis of the homopolymers stretches of 6 bp or longer using the BRCA HP v2.0 kit (Multiplicom) allows the exclusion of all variants detected in homopolymers ≥ 6 bp from the variant list reported by the AVA 2.7 software, increasing the specificity of the detection of* BRCA1* and* BRCA2* mutations to 99.99%.

### 3.2. Detection of* BRCA1* and* BRCA2* Mutations in a Cohort of 101 Patients with Inherited Breast and Ovarian Cancer

We next decided to implement our parallel pyrosequencing protocol and sequence analysis approach to screen for mutations in the* BRCA1* and* BRCA2* genes in a series of 101 patients with breast and ovarian cancer. Our objective was to further analyze the performance of massive parallel pyrosequencing in terms of number of sequences obtained per run, coverage uniformity, and number of variants detected as well as in the identification of pathogenic mutations responsible for the disease.

All samples were first analysed for mutations in the homopolymer stretches of >6 bp using the BRCA HP v2.0 kit. Three frameshift mutations were detected. Although they are not strictly located in the homopolymer stretch, they are within the fragments amplified by the BRCA HP v2.0 kit (Table 4). Sanger sequencing identified one deletion, one insertion, and a combined InDel (c.4030del6insC, c.5189dupA, and c.5722_5723delCT in the* BRCA2* gene).

The remaining 98 samples were distributed in 14 Junior runs in groups of seven samples. We decided to sequence seven samples per run instead of eight in order to increase the coverage per amplicon and to decrease the number of amplicons with low coverage (<38 reads).

The number of reads obtained per run was very variable. The reads that passed the quality filters ranged between 69455 reads and 150722 reads with an average of 99864 reads (±28215) (Table 3). As a result of the differences between the reads obtained per run, the average coverage per amplicon and most importantly the number of amplicons with less than 38 reads were also variable (Table 3). Depending on the run the number of amplicons with less than 38 reads ranged between 1 and 26.

In the total 14 GS Junior runs, we identified 14 patients with deleterious mutations of which 7 are frameshift mutations (one mutation was found in three different patients), 4 are nonsense, 2 are missense, and 1 is an in frame deletion that affects splicing (Table 4). All mutations were confirmed by Sanger sequencing discarding the presence of false positives. One* BRCA1* mutation, c.68_69delAG, was found in three different patients. This mutation accounts for the 30.4%* BRCA1* mutations in the Mediterranean area [22]. Although only found once in our series, mutations c.211A>G, c.5123C>A in* BRCA1*, and c.3264dupT in* BRCA2* are also considered recurrent in the Spanish population [22]. We have identified 5 novel mutations in our cohort. Mutation c.2900_2901dupCT in the* BRCA1* gene and mutations c.4030del6insC, c.5189dupA, c.8009delC, and c.9274delT in the* BRCA2* gene are mutations not described previously. In addition we detected 9 variants with unknown clinical significance (VUS). All of them are missense mutations except one located in an intronic sequence (c.68-7T>A in the* BRCA2* gene). Finally, large genomic deletions and duplications were screened using the MAQ assay, which consists in a semiquantitative PCR that amplifies all exons in the* BRCA1* and* BRCA2* genes together with control regions. We detected an exonic deletion that comprises exons 16 and 17 of the* BRCA1* gene. This mutation is predicted to produce an inframe deletion of 132 amino acids that disrupts the BRCT-N domain (p.Glu1559_Thr1691del) and it has been described to be deleterious by functional analysis [23].

## 4. Discussion

Molecular genetic testing of mutations in the* BRCA1* and* BRCA2* genes is currently performed using highly sensitive but labour-intensive direct Sanger sequencing of individual exons. The advances in sequencing technologies have increased the speed and efficiency of DNA testing and next generation platforms are becoming the standard in molecular genetic diagnosis.

Here, we have tested and implemented a method for the molecular analysis of the* BRCA1* and* BRCA2* genes based on massive parallel pyrosequencing of pooled* BRCA1* and* BRCA2* gene enriched samples.* BRCA1* and* BRCA2* exon enrichment has been performed by PCR amplification using the Multiplicom BRCA MASTR kit, which amplifies all* BRCA1* and* BRCA2* coding exons in 97 amplicons. PCR enrichment was chosen over a hybridisation based method because PCR enrichment has been shown to cover all the amplicons of interest and to provide less variation in coverage between regions [10]. In addition, PCR enrichment has also a lower cost and requires less DNA compared to hybridisation based methods. Currently, PCR based enrichment is chosen for molecular diagnosis when analysing few genes simultaneously.

We validated our approach in a cohort of 23 patients with previously characterised* BRCA1* and* BRCA2* mutations and polymorphisms. We detected all mutations and polymorphisms in both heterozygosity and homozygosity achieving 100% sensitivity and 97.35% specificity. To increase the specificity of the method the variants called in homopolymeric regions should be excluded. Because both* BRCA1* and* BRCA2* genes comprise homopolymeric stretches in their coding regions a complementary assay is then needed to screen for changes in homopolymers. We used the BRCA HP assay developed by Multiplicom which screens for deletions and insertions in all exonic homopolymers of 6 bp or longer. This allowed the exclusion from our final variant list all changes detected in homopolymeric regions of ≥6 nucleotides resulting in a specificity of 99.99%.

Other works have analysed the performance of pyrosequencing in the detection of mutations in the* BRCA1* and* BRCA2* genes using different approaches to obtain the* BRCA1* and* BRCA2* DNA library and using the 454 GS FLX and GS Junior platforms [15–18]. Here, we used a multiplex amplicon based assay which amplifies all* BRCA1* and* BRCA2* coding regions and exon-intron boundaries and attaches the MIDs and sequencing adaptors in a second PCR (BRCA MASTR, Multiplicom). Multiplex PCR has been demonstrated to result in higher coverage per amplicon compared to singleplex [15] or long PCR fragments [17] and allows the joint sequencing of seven samples in each run. Recently, Feliubadaló et al. [18] have developed and validated a workflow using the BRCA MASTR kit amplicon followed by 454 GS Junior pyrosequencing. Data analysis combines the use of the three types of software VIP, R, and AVA and numerous filters followed by visual inspection of fragments. Their workflow achieves a specificity of 99.99% and a sensitivity of 100% when adding the BRCA HP assay to detect insertions and deletions in homopolymeric regions. In contrast to Feliubadaló et al. [18] our data analysis is based exclusively on the AVA 2.7 software making it simpler and completely automated. The AVA2.7 software in contrast to previous versions is able to call small InDels and achieve a sensitivity of 100% in variant calling ([16] and this report). Using our filtering parameters in the AVA 2.7 software together with BRCA HP assay we achieve a specificity of 99.99% and a sensitivity of 100%.

It is recommended that mutations detected by NGS technologies be validated by Sanger sequencing in the context of molecular diagnostics. Here, all deleterious mutations and VUS detected in our cohort of 101 patients have been confirmed by Sanger sequencing. These results together with the ones obtained in our validation set show that when using massive parallel pyrosequencing only deleterious mutations detected in homopolymeric tracts should be confirmed by Sanger sequencing [16].

Analysis of the coverage in our series of 14 runs showed that the number of amplicons with less than 38x ranged from 1 to 26 (0.14–3.8%) of a total number of 679 amplicons sequenced per run. This means that seven samples can be screened in a single GS Junior run with more than 95% of sequences covered sufficiently to provide a minimum power of 99.9% to detect heterozygous mutations in at least 25% of the reads. We detected that the number of reads obtained per run was very variable. After carefully reviewing our whole procedure, we realised that the addition of a lower number of molecules of DNA library per bead in the emulsion PCR resulted in the higher number of reads that passed quality filtering. Taking into account this observation we are now increasing the number of samples per run, which will result in a lower cost per sample analysed. We have checked that the cost and time consuming per sample of our sequencing approach improves the overall cost (approximately 50% less) and makes the process faster compared to direct Sanger sequencing alone.

**Figure 1:**
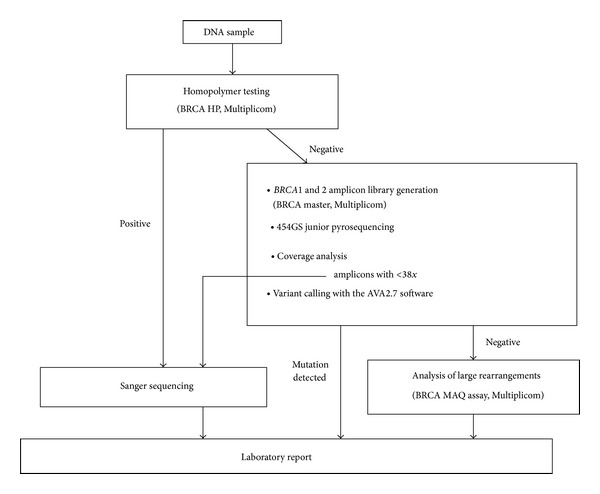
Proposed workflow using massive parallel pyrosequencing for analysing* BRCA1* and* BRCA2* genes.

Our proposed workflow to screen for mutations in the* BRCA1* and* BRCA2* genes consists first in the use of the* BRCA1* and* BRCA2* homopolymer assay (BRCA HP) followed by massive parallel sequencing with the 454 GS Junior sequencer and using the BRCA MASTR amplicon kit to generate the patient libraries. Coverage and variant calling is done using the AVA 2.7 software. Amplicons with low coverage should be Sanger sequenced. Finally, large rearrangements in the* BRCA1* and* BRCA2* genes are detected using the BRCA MAQ kit (Figure 1). Using our validated workflow, we have identified 18 deleterious mutations in 101 patients (17,8%) which is in accordance with the prevalence of* BRCA1* and* BRCA2* mutations reported in the Spanish hereditary breast and ovarian cancer population. In addition, we have detected 10 VUS, nine of which are unique and two of them have not been previously reported.

## 5. Conclusions 

We show here that massive parallel pyrosequencing can be used as a diagnostic strategy to test for* BRCA1* and* BRCA2* mutations meeting very stringent sensitivity and specificity parameters and could be used in diagnostic laboratories replacing traditional Sanger sequencing.

## Supplementary Material

Supplementary Table 1. shows the frequency of detection of each variant both in the forward and reverse reads.
Supplementary Table 2. shows the false positives detected by the AVA2.7 software.

## Figures and Tables

**Table 1:** Summary of sequencing runs and coverage results of the validation set.

	Run 1	Run 2	Run 3
	BRCAMASTRv1.2	BRCAMASTRv2.0	BRCAMASTRv2.0
Samples	7	8	8
Amplicons	169	94	94
Passed filter reads	118006	78777	100771
Mapped reads	113374	78698	100344

Coverage (number of reads/amplicon)			
Minimum	19	13	17
Mean	96,98	102,7	132,27
Maximum	277	445	414
Standard deviation coverage	35,8	46,92	70,51
Amplicons <38 reads (%)	32 (2.7)	32 (4.2)	13 (1.72)

**Table 2:** Pathogenic mutations in the validation set tested for the evaluation of the AVA 2.7 software.

Variant HGVS	Gene	Variant freq. % (number of reads)	
		forward	reverse
c.70_71insTGTC	*BRCA1 *	55.88 (68)	59.32 (59)
c.1121-1123delCACinsT	*BRCA1 *	35 (60)	49.25 (67)
c.1961delA	*BRCA1 *	38.68 (106)	31.07 (103)
c.2921T>A (p.L974X)	*BRCA1 *	51.16 (43)	36.21 (58)
c.3767_3768delCA	*BRCA1 *	26 (50)	54.69 (64)
c.3770-3771delAG	*BRCA1 *	50 (50)	47.46 (59)
c.4107-4110dupATCT	*BRCA1 *	54.24 (59)	51.85 (54)
c.5123C>A	*BRCA1 *	52.38 (42)	56.25 (48)
c.1842dupT	*BRCA2 *	56.00 (25)	55.17 (29)
c.5350-5351delAAinsT	*BRCA2 *	48.92 (139)	54.08 (98)
c.6275-6276delTT	*BRCA2 *	47.41 (116)	38.13 (139)
c.7617+1G>A	*BRCA2 *	34.78 (23)	48.28 (29)
c.8946delA^a^	*BRCA2 *	100 (52)	44.44 (36)
c.9026_9030delATCAT	*BRCA2 *	50.94 (53)	40.54 (37)

^a^Mutation located in a homopolymeric region.

**Table 3:** Summary of reads obtained and coverage results in 14 GS Junior runs.

Run	1	2	3	4	5	6	7	8	9	10	11	12	13	14
Passed filter reads	113809	69455	82287	73127	88687	138625	72728	110271	90416	136237	69811	121332	80589	138238
Mapped reads	113445	69184	81904	72835	87529	137794	72522	109691	89982	135982	69613	12107	77080	138190

Coverage (number of reads/amplicon)														
Mean	213,28	106,88	127,26	113,81	134,03	202,36	110,44	167,54	137,11	207,24	106,07	184,66	117	196.9
Minimum	5	29	22	34	19	24	26	27	31	7	23	21	11	8
Maximum	750	377	394	417	1560	1455	823	556	439	663	383	728	509	734
Amplicons <38 reads (%)	7	13	10	1	20	2	20	2	1	10	8	8	26	18

**Table 4:** Summary of BRCA1 and BRCA2 pathogenic mutations and variants of unknown significance (VUS) detected using our proposed workflow.

Variant HGVS	Gene	Detected with assay	Clinical significance	
c.68_69delAG (p.Glu23Valfs^∗^16)	*BRCA1 *	NGS	Pathogenic	Spanish recurrent mutation
c.211A>G (p.Arg71Gly)	*BRCA1 *	NGS	Pathogenic	Spanish recurrent mutation
c.2410C>T (p.Gln804^∗^)	*BRCA1 *	NGS	Pathogenic	Reported
c.2900_2901dupCT (p.Pro968Leufs^∗^32)	*BRCA1 *	NGS	Pathogenic	Novel
c.3406C>A p.(Pro1136Thr)	*BRCA1 *	NGS	VUS	Novel
c.3708T>G (p.Asn1236Lys)	*BRCA1 *	NGS	VUS	Reported
c.4935G>C (p.Arg1645Ser)	*BRCA1 *	NGS	VUS	Reported
c.5078_5080delCTG (p.1692del26)	*BRCA1 *	NGS	Pathogenic	Reported
c.5123C>A (p.Ala1708Glu)	*BRCA1 *	NGS	Pathogenic	Spanish recurrent mutation
Δ Exons 16/17	*BRCA1 *	MAQ	Pathogenic	Reported

c.68-7T>A	*BRCA2 *	NGS	VUS	Reported
c.754G>A (p.Asp252Asn)	*BRCA2 *	NGS	VUS	Novel
c.3264dupT (p.Gln1089Serfs^∗^8)	*BRCA2 *	NGS	Pathogenic	Spanish recurrent mutation
c.4030del6insC (p.Asn1344Hisfs^∗^5)	*BRCA2 *	HP	Pathogenic	Novel
c.4316C>A (p.Ala1439Asp)	*BRCA2 *	NGS	VUS	Reported
c.4681C>A (p.His1561Asn)	*BRCA2 *	NGS	VUS	Reported
c.4965C>A (p.Tyr1655^∗^)	*BRCA2 *	NGS	Pathogenic	Reported
c.5189dupA (p.Asn1730Lysfs^∗^12)	*BRCA2 *	HP	Pathogenic	Novel
c.5722_5723delCT (p.Leu1908Argfs^∗^1)	*BRCA2 *	HP	Pathogenic	Reported
c.6215C>G (p.Ser2072Cys)	*BRCA2 *	NGS	VUS	Reported
c.6613G>A (p.Val2205Met)	*BRCA2 *	NGS	VUS	Reported
c.7180A>T (p.Arg2394^∗^)	*BRCA2 *	NGS	Pathogenic	Reported
c.7480C>T (p.Arg2494^∗^)	*BRCA2 *	NGS	Pathogenic	Reported
c.8009delC (p.Ser2670Trpfs^∗^2)	*BRCA2 *	NGS	Pathogenic	Novel
c.9274delT (p.Tyr3092Ilefs^∗^11)	*BRCA2 *	NGS	Pathogenic	Novel

NGS: next generation sequencing; HP: homopolymer; MAQ: multiple amplicon quantification.
