# Supplementary material for: Genetic Testing in Hereditary Breast and Ovarian Cancer Using Massive Parallel Sequencing
Source: Biomed Res Int. 2014 Jun 26;2014:542541. doi: 10.1155/2014/542541 (PMC4098986; doi:10.1155/2014/542541)
Supplement: Supplementary file 1 — Supplementary Table 1. shows the frequency of detection of each variant both in the forward and reverse reads. Supplementary Table 2. shows the false positives detected by the AVA2.7 software. [file 542541.f1.pdf]

Supplementary Table 1. Frequency variant detection by the AVA 2.7 software.

Variant HGVS	Gene	Pathogenicity	Variant freq (nº of reads; MID) Run1		Variant freq (nº of reads; MID) Run2		Variant freq (nº of reads; MID) Run3	
			forward	reverse	forward	reverse	forward	reverse
c.1-134C>T	BRCA1	POL	62.16 (37;2)	46.34(41;2)	100 (68;1)	100 (59;1)	56.67(159;5)	48.94(94;5)
c.70_71insTGTC	BRCA1	PATH	Not present		55.88(68;1)	59.32(59;1)	Not present	
c.442-34C>T	BRCA1	POL	58.82(34;8)	62.86(35;8)	51.72(29;4)	70.27(37;4)	60.98(82;6)	44.23(52;6)
c.548-58delT[a]	BRCA1	POL	100(67;1)	90,74(108;1)	35(40;2)	76,36(55;2)	70,37(27;4)	95,35(43;4)
c.591C>T	BRCA1	POL	56.52(23;2)	43.75(32;2)	Not present		53.57(28;1)	41.67(12;1)
c.1121-1123delCACinsT	BRCA1	PATH	35 (60;8)	49.25 (67;8)	Not present		Not present	
c.1961delA[a]	BRCA1	PATH	38.68(106;6)	31.07(103;6)	Not present		Not present	
c.2077G>A	BRCA1	POL	43.33(90;6)	44.23(104;6)	Not present		Not present	
c.2082C>T	BRCA1	POL	52.33(86:8)	54.81(104;6)	100 (45;1)	100 (51;1)	100.00(47;2)	100.00(48;2)
c.2311 T>C	BRCA1	POL	34.02(97;8)	46.25(80;8)	100 (82;1)	100 (108;1)	49.35(154;3)	51.20(166;3)
c.2612C>T	BRCA1	POL	99.00(100;4)	98.75(80;4)	98,29 (117;1)	100 (138;1)	60.71(112;4)	52.14(117;4)
c.2634A>G	BRCA1	POL	Not present		Not present		45.24(126;8)	53.79(132;8)
c.2921T>A (p.L974X)	BRCA1	PATH	Not present		51.16 (43:2)	36.21(58.2)	Not present	
c.3113A>G	BRCA1	POL	99.15(117;6)	99.06(106;6)	47.92(48;6)	55.84(77;4)	48.70(154;7)	50.00(170;7)
c.3119G>A	BRCA1	POL	Not present		54.17(24;6)	47.62(42;6)	48.84(43;4)	59.57(47;4)
c.3548A>G	BRCA1	POL	52.14(117;2)	47.06(102;2)	100.00(73;5)	100.00(86;5)	41.46(82;3)	68.00(75;3)
c.3767_3768delCA	BRCA1	PATH	Not present		Not present		26 (50;3)	54.69 (64;3)
c. 3770-3771delAG	BRCA1	PATH	50 (50;4)	47.46 (59;4)	Not present		Not present	
c.4097-141A>C	BRCA1	POL	Not present		50.54(93;7)	50.00(88;7)	44.97(149;3)	47.53(162;3)
c.4107-4110dupATCT	BRCA1	PATH	54.24(59;5)	51.85(54;5)	Not present		Not present	
c.4113G>A	BRCA1	POL	Not present		Not present		51.61(155;4)	53.42(146;4)
c.4308T>C	BRCA1	POL	100.00 (97;6)	100.00(130;6)	50.00(32;8)	48.94(47;8)	100.00(67;2)	100.00(52;2)
c.4485-63C>G	BRCA1	POL	Not present		38.46(78;7)	50.00(102;7)	41.67(48;1)	43.75(48;1)
c.4837A>G	BRCA1	POL	48.48(66;2)	52.83(53;2)	51.85(162;4)	51.11(180;4)	47.89(71;3)	38.64(88;3)
c.4987-68A>G	BRCA1	POL	Not present		48.89(90;4)	51.22(123;4)	52.00(75;5)	49.40(83;5)
c.4987-92A>G	BRCA1	POL	Not present		48.89(90;4)	51.22(123;4)	52.00(75;5)	50.60(83;5)
c.5074+6C>G	BRCA1	VUS	Not present		49.49(99;5)	49.63(135;5)	Not present	
c.5123C>A	BRCA1	PATH	52.38(42;2)	56.25(48)	Not present		50.00(58;1)	33.93(56;1)
c.5152+66G>A	BRCA1	POL	45.24(42;2)	41.67(48;2)	25.00(12;6)	50.00(32;6)	52.94 (102;6)	64.37 (87;6)
c.5467+9C>A	BRCA1	VUS	58.70(46;4)	43.48(46;4)	Not present		Not present	

c.1-26G>A	BRCA2	POL	49.30(71;7)	51.28(78;7)	53.03(66;8)	38.46(52;8)	44.44(54;4)	42.86(56;4)
c.425+67A>C	BRCA2	POL	Not present		Not present		54.39(57;8)	53.85(52;8)
c.426-89T>C	BRCA2	POL	Not present		Not present		49.18(61;8)	45.07(71;8)
c.631+183T>A	BRCA2	POL	Not present		60.53(38;3)	57.14(14;3)	49.18(61;1)	42.31(52;1)
c.681+56C>T	BRCA2	POL	Not present		52.08(48;1)	40.43(47;1)	34.21(38:5)	52.38(42:5)
c.865A>C	BRCA2	POL	Not present		Not present		44.12(34;1)	60.53(38;1)
c.1114A>C	BRCA2	POL	45.45(22;7)	36.84(19;7)	59.52 (42;1)	76.47 (17;1)	38.24(34;2)	45.16(31;2)
c.1365A>G	BRCA2	POL	Not present		Not present		42.11(38;1)	62.07(58;1)
c.1842dupT	BRCA2	PATH	Not present		Not present		56.00(25;5	55.17(29;5)
c.2229T>C	BRCA2	POL	Not present		Not present		39.13(23;1)	42.42(33;1)
c.2803G>A	BRCA2	POL	54.61(141;7)	56.13(155;7)	Not present		Not present	
c.2971A>G	BRCA2	POL	Not present		Not present		54.26(94;1)	563.22(87;1)
c.3170A>G	BRCA2	POL	Not present		Not present		53.61(166;7)	52.71(203;7)
c.3396A>G	BRCA2	POL	63.33(30;7)	57.14(35;7)	51.22(82;3)	44.32(88;3)	100.00(69;4)	100.00(94;4)
c.3807T>C	BRCA2	POL	100.00(80;6)	97.44(78;6)	45.00(60;5)	46.67(45;5)	51.69(89;3)	33.78(74;3)
c. 5350-5351delAAinsT	BRCA2	PATH	48.92 (139;7)	54.08 (98;7)	Not present		Not present	
c.5640T>G	BRCA2	POL	Not present		Not present		49.02(51;3)	47.3(74;3)
c.6275-6276delTT	BRCA2	PATH	47.41(116;1)	38.13(139;1)	Not present		Not present	
c.6841+79delTTAA	BRCA2	POL	99.06(106;5)	98,08(104;5)	63.21(49;3)	41.94(31;3)	100(31;4)	100(24;4)
c.7008-62A>G	BRCA2	POL	36.11(36;7)	36.11(36;7)	Not present		Not present	
c.7150C>A	BRCA2	POL	Not present		Not present		49.02(51;3)	47.30(74;3)
c.7242A>G	BRCA2	POL	54.72(106;1)	56.38(94;1)	Not present		45.71(105;6)	51.18(127;6)
c.7617+1G>A	BRCA2	PATH	Not present		Not present		34,78(23)	48.28(29)
c.7806-14T>C	BRCA2	POL	48.57(35;1)	50.00(40;1)	44.44(54;4)	60.00(45;4)	100.00(56;5)	100.00(35;5)
c.8149G>T	BRCA2	POL	Not present		52.94(34;8)	29.03(31;8)	Not present	
c.8755-66T>C	BRCA2	POL	Not present		43.59(39;4)	60.00(32;4)	100,00(28;5)	100,00(28;5)
c.8851G>A	BRCA2	POL	Not present		Not present		48.48(66;8)	56.36(55;8)
c.8946delA[a]	BRCA2	PATH	Not present		Not present		100 (52;6)	44.44(36;6)
c.9026_9030delATCAT	BRCA2	PATH	Not present		Not present		50.94 (53;2)	40.54 (37;2)
c.9257-16T>C	BRCA2	POL	62.79(43;1)	45.00(40;1)	Not present		Not present	
c.9976A>T	BRCA2	POL	30.43(23;1)	40.74(27;1)	Not present		Not present	
c.10362A>C	BRCA2	POL	Not present		64.00(25;7)	58.54(41;7)	58.33(24;7)	39.20(28;7)

[a] variants located in homopolimeric regions

Supplementary Table 2. False positives detected by the AVA2.7 software.

Type of variant	Gene	Hompolimeric region	Sequence homopolimer	Run	Location
c.81-18delC	BRCA1	Yes	6C	Run2/3	Intron 2
c.302 -138delA	BRCA1	Yes	8A	Run2/3	Intron 6
c.329delA	BRCA1	Yes	6A	Run3	Exon 7
c.441+40delT	BRCA1	Yes	4T	Run2/3	Intron 7
c.441+50_51delTT	BRCA1	Yes	10T	Run2	Intron 7
c.441+51del14	BRCA1	Yes	10T	Run2/3	Intron 7
c.441+51delT	BRCA1	Yes	10T	Run2	Intron 7
c.441+63_64delTT	BRCA1	Yes	12T	Run2	Intron 7
c.441+64del4	BRCA1	Yes	12T	Run2	Intron 7
c.441+64del	BRCA1	Yes	12T	Run2/3	Intron 7
c.548-58delTT	BRCA1	Yes	7T	Run2	Intron 8
c.548-58delT	BRCA1	Yes	7T	Run1/2/3	Intron 8
c.548-49delT	BRCA1	Yes	4T	Run2/3	Intron 8
c.548-18delT	BRCA1	Yes	6T	Run2	Intron 8
c.671-49delT	BRCA1	Yes	6T	Run2	Intron 10
c.1016delA	BRCA1	Yes	7A	Run2/3	Exon 11
c.1961delA	BRCA1	Yes	8A	Run1	Exon 11
c.68-16delT	BRCA2	Yes	10T	Run1/2/3	Intron 2
c.476-15delT	BRCA2	Yes	7T	Run1/2/3	Intron 5
c.632-61delT	BRCA2	Yes	6A	Run1/2/3	Intron 7
c.1593delA.	BRCA2	Yes	6A	Run1/2	Exon 10
c.1813delA.	BRCA2	Yes	8A	Run1/2/3	Exon 10
c.1909+22delTTT	BRCA2	Yes	11T	Run1	Intron10
c.1909+22delTT	BRCA2	Yes	11T	Run1/2/3	Intron10
c.1909+22delT	BRCA2	Yes	11T	Run1/2/3	Intron10
c.2835delA	BRCA2	Yes	6A	Run2	Exon 11
c.2957delA	BRCA2	Yes	7A	Run2	Exon 11
c.4169delT	BRCA2	Yes	6T	Run1/2/3	Exon11
c.4593delA	BRCA2	Yes	6A	Run1/2/3	Exon11
c.5351delA	BRCA2	Yes	6A	Run1	Exon11
c.7618-27delT	BRCA2	Yes	6T	Run2/3	Intron15
c.8940delA	BRCA2	Yes	7A	Run1/2/3	Exon22
c.9097delA	BRCA2	Yes	8A	Run1/3	Exon23
c.9256+28delT	BRCA2	Yes	8A	Run1/2/3	Intron24
c.9257-17delT	BRCA2	Yes	8T	Run1/2/3	Intron24
c.9649-53delT	BRCA2	Yes	6T	Run3	Intron 26
c.9945delA	BRCA2	Yes	6A	Run1	Exon27
